# Evolutionary status of the invasive muskrat *Ondatra zibethicus* revealed by complete mitochondrial genome

**DOI:** 10.1080/23802359.2020.1719931

**Published:** 2020-02-03

**Authors:** Lina Zhang, Han Zhang, Yan Hua

**Affiliations:** a Eco-Engineering Department, Guangdong Eco-Engineering Polytechnic, Guangzhou, Guangdong, China;; b College of Wildlife and Protected Areas, Northeast Forestry University, Harbin, Heilongjiang, China;; c Guangdong Provincial Key Laboratory of Silviculture, Protection and Utilization, Guangdong Academy of Forestry, Guangzhou, China

**Keywords:** mtDNA genome, evolutionary relationship, muskrat *Ondatra zibethicus*

## Abstract

The muskrat *Ondatra zibethicus* is native to North America, However it has successfully dispersed into most areas of northeast China in the past decades, which may lead to potential impact on endemic eco-system. We determined and annotated the whole mtDNA genome of the muskrat *O. zibethicus* to better understand the evolutionary relationship of this invasive species with other *Rodentia* distributed in China. The complete mitogenome is 16,351 bp in length, includes 13 protein-coding genes, 22 tRNA genes, 2 rRNA genes, and a control region. We built the phylogenetic tree of muskrat and other 11 most closely related *Rodentia species.*

The muskrat (*Ondatra zibethicus*) is a highly adaptable rodent native to North America (Simpson and Boutin 1993; Greenhorn et al. 2017). Due to natural diffusion and the rise of fur breeding, the muskrat’s proliferation in China has been greatly promoted. From 1927, the former Soviet Union began to release muskrats in areas such as Amur River Basin. In the 1950s, muskrats were widespread in the waters of China’s Heilongjiang border region. In order to develop the fur animal industry, the Chinese government started stocking muskrat. There are also some people in Heilongjiang Province of China who have begun to raise them.

Alien species invasion is one of the serious global problems facing the ecosystem (Rotherham and Lambert 2012; Jo et al. 2017). The ecological cost is the loss of native species diversity and species extinction, which constitute an important threat to the conservation and sustainable utilization of biological diversity and the human living environment (Danell 1979; Bos and Ydenberg 2011). With the increasing frequency of introduction and trade between regions in the global animal breeding industry, the artificial introduction has become one of the important channels for the invasion of foreign species. Although the Chinese government attaches great importance to the protection of species, the genetic evolution of muskrat remains unclear in Northeast China, and its population status and genetic evolution also are unclear.

In order to find out the origin and genetic evolution relationship of muskrat, we collected feral muskrat samples in the Songhua river basin around Harbin city, Heilongjiang province, China (N45.808596°, E126.584059°). The muskrat samples were accidentally caught by fishermen. To provide a reference for scientific management and effective prevention and control of muskrat invasion, we conducted genetic research with samples. And as part of the research, we determined and annotated the complete mitochondrial genome of an invasive muskrat, *O. zibethicus*, using 13 pairs of primers (Saccone et al. 1999). The muscle tissue used for DNA extraction and analysis was sampled from and the specimen is stored in the Feline Research Center of Chinese State Forestry Administration, China. The sample number is NEFU-F2-19-75.

In addition, to reveal the phylogenetic relationship of this species, we reconstructed the complete mitochondrial genome-based neighbor-joining tree of the muskrat *O. zibethicus* and other 11 *Rodentia* species (Gibson et al. 2004) distributed in China with Kimura 2-parameter model using MEGA version 7 (Kumar et al. 2016), and the tree was tested with 1000 Bootstrap replications. The complete mitogenome of *O. zibethicus* is 16,351 bp in length (GenBank accession number MN 485774), which is made up of 13 protein-coding genes, 2 rRNA genes, 22 tRNA genes, and a control region. The overall base composition is A: 34.5%, T: 27.6%, C: 25.3%, and G: 12.7%, with a much higher A + T content.

The phylogenetic neighbor-joining tree is shown in Figure 1 and we can see from the figure that the muskrat *O. zibethicus* is closest to the genus *Eothenomys* with high support. This study can provide the basis for the genetic evolution of muskrat.

**Figure 1.**
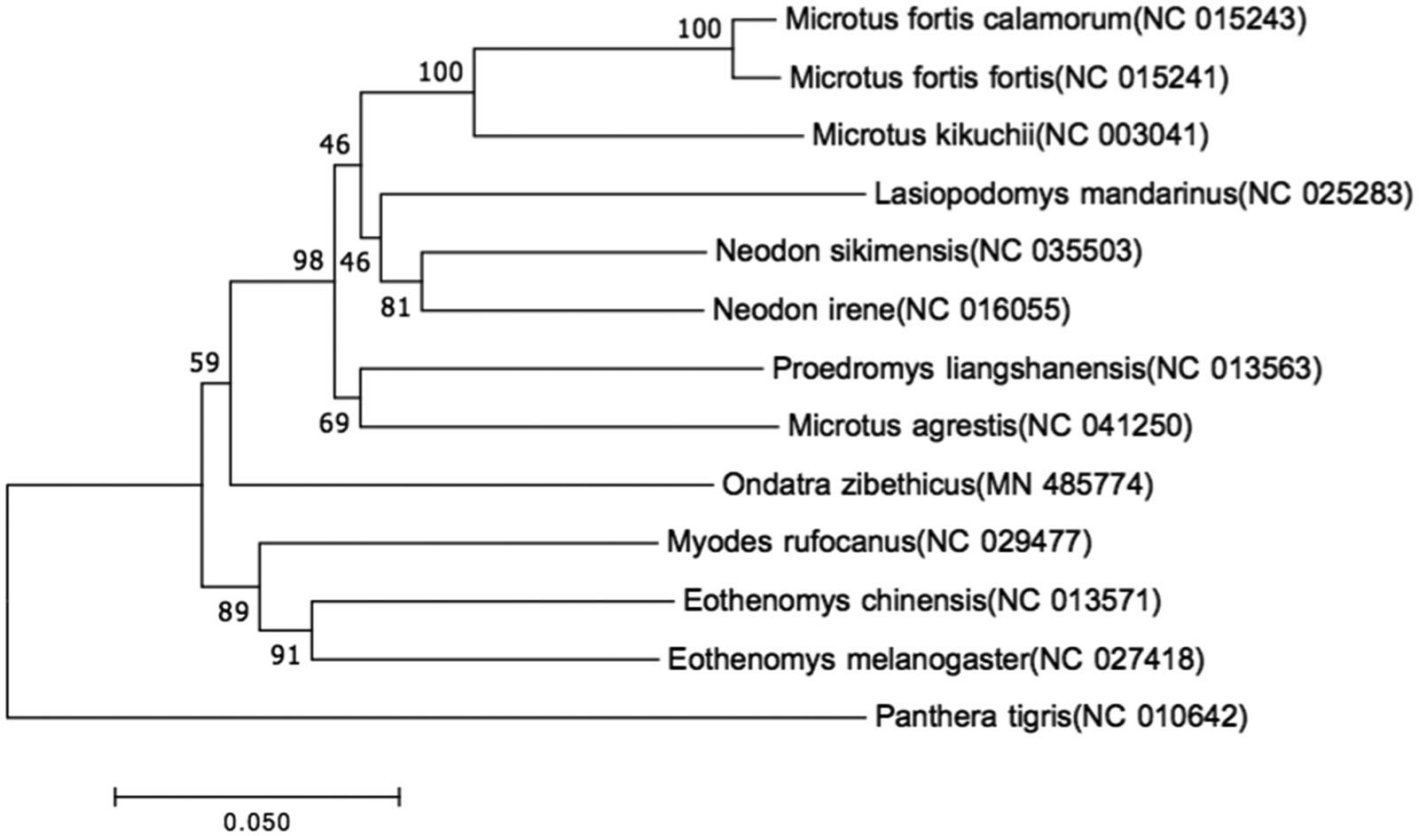
Neighbor-joining phylogenetic tree of muskrat and other 11 species of *Rodentia* constructed by MEGA Version 7.0. *COI* (Boykin et al. 2007) gene sequence of other 11 species of *Rodentia* are downloaded from NCBI and the GenBank accession numbers are given in the bracket after the species name.
